# Nutritional Screening and Assessment in Different Populations

**DOI:** 10.3390/nu17091525

**Published:** 2025-04-30

**Authors:** Aránzazu Aparicio, Mᵃ del Carmen Lozano-Estevan

**Affiliations:** 1 Valornut Research Group, Department of Nutrition and Food Science, Faculty of Pharmacy, Complutense University of Madrid, 28040 Madrid, Spain; araparic@ucm.es; 2 San Carlos Health Research Institute (IdISSC), 28040 Madrid, Spain

## 1. Introduction

Nutritional assessment in different populations is a key strategy in both clinical practice and public health, and systematic nutritional screening helps detect early indicators of malnutrition, which may include undernutrition, obesity, or specific micronutrient deficiencies. Early identification makes more targeted and effective dietary and healthcare interventions possible. Additionally, identifying nutritional disparities based on age, sex, physiological status, or sociocultural context facilitates the classification of risk factors and priority needs. This process informs the design of preventive policies and tailored nutritional care programs for vulnerable groups.

This Special Issue of *Nutrients*, entitled “Nutritional Screening and Assessment in Different Populations”, brings together scientific contributions conducted across various settings—from indigenous communities and pediatric populations to individuals with chronic conditions and young adults. Each study explores specific screening methodologies or investigates associations between nutritional status and relevant clinical or epidemiological variables. The key findings of the articles featured in this volume are summarized below, emphasizing their relevance to the overarching theme and their contribution to the advancement of nutritional science.

## 2. The Development and Synthesis of Contributions in This Special Issue

Nutritional assessment in socioeconomically disadvantaged populations is critically important. Ravula et al. (Contribution 1) investigated the prevalence of malnutrition (stunting, underweight, or thinness) among adolescent girls in indigenous communities in Telangana, India. Their results show that only 13% of the 695 participants had a normal nutritional status, with 87% exhibiting signs of chronic or acute malnutrition. The underlying factors include structural issues such as poverty, lack of education, and inadequate access to sanitation and health resources. These findings underscore the urgent need for multisectoral interventions and systematic nutritional surveillance in historically marginalized groups. The early detection of nutritional imbalances is essential to preventing harmful consequences in terms of growth and development [1,2].

Even young, ostensibly healthy populations can harbor significant nutritional deficits. Dimas-Benedicto et al. (Contribution 2) explored the association between iron intake, body iron stores, and cognitive performance in university students. They found a strong association between iron insufficiency and reduced cognitive performance, particularly in women. Their study emphasizes the importance of screening for micronutrient deficiencies in academic settings and that identifying “hidden hunger” during periods of heightened academic demand enables tailored nutritional interventions aimed at optimizing brain health and academic success [3,4]. Thus, this study underscores that nutritional examination should not be confined to traditionally high-risk groups but can be valuable for wider segments of the general population.

The introduction of processed foods with high protein content has generated substantial interest among researchers. Ortega et al. (Contribution 3) examined the impact of consuming protein-enriched processed foods on overall dietary quality and health. Through survey data and a detailed examination of nutritional labeling, the authors noted an increasing intake of such products, often driven by perceived nutritional benefits. However, they warn that excessive protein consumption may disrupt dietary balance and displace other essential nutrients. Raising consumer awareness, strengthening advertising regulations, and nutritional education are necessary to discourage unbalanced dietary behaviors.

Systematic assessment methods tailored to early childhood can prevent long-term complications [4,5]. Arias et al. (Contribution 4) developed and validated a nutritional screening instrument specifically targeted at Hispanic preschoolers, achieving promising sensitivity and specificity. When implemented in community and educational contexts, it could facilitate systematic detection of undernutrition and overweight in children aged 3 to 5 years. This opens opportunities for culturally adapted early interventions.

Similarly, Ruder and Lohse (Contribution 5) proposed an index grounded in Satter’s Division of Responsibility in Feeding (sDOR.2-6y™) to evaluate the familial context shaping children’s eating behaviors (Contribution 5). They observed that sDOR.2-6y™ scores can identify nutritional risk even in the absence of abnormal BMI or household food insecurity. Including behavioral and psychosocial dimensions in screening, rather than relying solely on anthropometric or biochemical indicators, provides a comprehensive understanding of children’s nutritional status [6].

Both undernutrition and obesity may coexist in rare or chronic diseases. Wernio et al. (Contribution 6) reported that approximately half of children with Duchenne Muscular Dystrophy present some degree of nutritional alteration, either excess or deficiency. Their data confirm the need for the periodic monitoring of body composition and muscle functionality to reduce complications. Their Pediatric Nutrition Screening Tool (PNST) has emerged as a rapid and effective approach for detecting nutritional risk, provided it is supplemented with thorough clinical and dietary evaluations [7].

Moreover, Halilagic et al. (Contribution 7) conducted a systematic review of methods of assessing energy expenditure in Prader–Willi syndrome, which progresses from hypotonia to severe obesity. Predictive equations of energy expenditure often show limitations in this context, and do not typically account for the atypical body composition, reduced muscle mass, and lower basal metabolic rate, observed in individuals with Prader–Willi syndrome [1]. Therefore, measurement techniques such as indirect calorimetry offer a more accurate assessment, ensuring appropriate dietary prescriptions and continuous monitoring of nutritional status [3].

In hospitalized patients, malnutrition may exacerbate morbidity and mortality. García-Moreno et al., (Contribution 8) explored the utility of C-reactive protein–prealbumin and C-reactive protein–albumin ratios as markers encompassing inflammation and visceral protein status, aimed at identifying malnutrition and predicting clinical outcomes. Their findings suggest that elevated values for these ratios are associated with a higher in-hospital mortality risk, while low levels may denote severe undernutrition [3]. Although these markers do not replace traditional indicators, they could strengthen nutritional screening in hospital settings and support more timely interventions [6].

## 3. Conclusions

The studies compiled in this Special Issue of *Nutrients* reaffirm the indispensable role of nutritional screening and assessment in identifying specific risks and informing interventions targeted at population-wide health improvements. Across different contexts—ranging from indigenous communities with structural disadvantages to individuals with complex genetic disorders, as well as preschoolers and university populations—the early identification of nutritional imbalances, alongside the objective measurement of clinical or behavioral markers, enables more effective preventive or therapeutic strategies.

Accordingly, nutritional assessment must be regarded as a cross-cutting element, built upon robust scientific criteria and aligned with the sociocultural realities of each cohort. The articles in this volume present both methodological insights and empirical evidence underscoring the centrality of nutritional evaluation in optimizing interventions, guiding public health policy, and advancing nutritional research.

## 4. Recommendations and Future Perspectives

- Systematic Implementation of Screening: Incorporating culturally adapted and validated screening instruments across healthcare levels (primary, specialized, community) may enhance the early detection of malnutrition, facilitating referral to specialized care or the provision of appropriate dietary support for at-risk individuals.
- Multidisciplinary Collaboration: Coordination among dietitians, pediatricians, family physicians, nurses, and social workers is essential to maximize the impact of nutritional assessment. Such integrated practice enables the identification of economic, educational, psychological, and cultural factors directly influencing dietary intake and nutritional status.
- Strengthening Nutrition Education: Disseminating dietary guidelines and delivering training in balanced eating may enhance the effectiveness of screening. Empowering the public with basic knowledge of dietary balance and nutritional variety could reduce both micronutrient deficiencies and obesity, fostering more informed food choices.
- Utilization of Complementary Tools: Integrating clinical, anthropometric, biochemical, and behavioral indicators (e.g., surveys of eating patterns or food security questionnaires) can significantly bolster diagnostic accuracy. This holistic approach generates a more complete understanding of an individual’s environment and thus refines decision-making in healthcare.
- Longitudinal Research and Ongoing Validation: Future prospective studies are needed to elucidate the trajectory of malnutrition and the long-term efficacy of nutritional interventions. Moreover, the continuous validation of screening tools in various age groups, geographical areas, and clinical conditions is recommended, aiming to establish consensus-based protocols.

In sum, the evidence presented herein highlights the vital importance of embedding nutrition assessment in routine practice and regarding nutrition as a transversal factor decisively influencing health and well-being. Adopting accurate and culturally appropriate screening strategies, reinforcing professional training, and empowering the general population in nutritional matters stand out as strategic cornerstones to successfully address emerging challenges in clinical nutrition and public health.
